# A Case of Takotsubo Cardiomyopathy in a 65-Year-Old Woman Triggered by Emotional Stress

**DOI:** 10.7759/cureus.73533

**Published:** 2024-11-12

**Authors:** Malak Bader, Manar Mubarak, Sara Ali, Zahra Hasan, Njood Alsudairy

**Affiliations:** 1 General Practice, Salmaniya Medical Complex, Manama, BHR; 2 College of Medicine, Wenzhou Medical University, Wenzhou, CHN; 3 College of Medicine, Southeast University, Nanjing, CHN; 4 College of Medicine, National University of Science and Technology, Muscat, OMN; 5 Radiology, The Second Jeddah Health Cluster, Jeddah, SAU

**Keywords:** acute coronary syndrome, broken heart syndrome, catecholamines, echocardiography, postmenopausal women, stress-induced cardiomyopathy, takotsubo cardiomyopathy

## Abstract

Takotsubo cardiomyopathy (TCM), also known as stress-induced cardiomyopathy or “broken heart syndrome,” is a transient cardiac condition that often mimics acute coronary syndrome (ACS) but lacks obstructive coronary artery disease. TCM primarily affects postmenopausal women and is often precipitated by physical or emotional stress. This report presents a case of TCM in a 65-year-old woman, emphasizing the diagnostic challenges and clinical management required to distinguish TCM from ACS. A 65-year-old woman presented to the emergency department with sudden chest pain and dyspnea following a recent emotional stressor. Her initial evaluation, including an ECG showing ST-segment elevation and mildly elevated troponin, suggested ACS. However, coronary angiography revealed no significant stenosis, and echocardiography demonstrated apical ballooning with preserved basal segment function, confirming a diagnosis of TCM. She was treated supportively with beta-blockers and an ACE inhibitor. Her hospital course was stable, and follow-up echocardiography showed normalization of left ventricular function, consistent with full recovery. This case contributes to the understanding of TCM, emphasizing the need for awareness in clinical practice to promptly diagnose and manage TCM effectively. Enhanced recognition of its triggers, pathophysiology, and diagnostic criteria can improve patient outcomes and reduce the risk of recurrence.

## Introduction

Takotsubo cardiomyopathy (TCM), also known as stress-induced cardiomyopathy or “broken heart syndrome,” is an acute and often reversible cardiac condition that mimics myocardial infarction but typically occurs without obstructive coronary artery disease [1,2]. First described in Japan in 1990, this syndrome is characterized by transient left ventricular dysfunction, usually affecting the apical and mid-ventricular segments, with hyperkinesis of the basal segments. The classic "apical ballooning" seen on echocardiography or ventriculography resembles a "takotsubo," a Japanese pot used to trap octopuses, giving the syndrome its name [1,2].

TCM predominantly affects postmenopausal women, accounting for approximately 90% of cases, and is often precipitated by intense physical or emotional stress. The underlying pathophysiology is not fully understood but is believed to involve a surge of catecholamines that lead to myocardial stunning [1-3]. Clinical presentation frequently mimics acute coronary syndrome (ACS), with symptoms such as chest pain, dyspnea, and elevated cardiac biomarkers. Electrocardiographic changes, particularly ST-segment elevations and T-wave inversions, further complicate differentiation from myocardial infarction [2,3].

Although prognosis is generally favorable with supportive care, TCM can lead to complications, including heart failure, arrhythmias, and, rarely, cardiogenic shock. Prompt recognition and differentiation from myocardial infarction are essential for optimal management and reducing the risk of recurrence [3,4].

## Case presentation

A 65-year-old woman presented to the emergency department with acute onset chest pain and shortness of breath that began suddenly while she was doing light physical activity at home. She described the chest pain as a pressing, tight sensation centered in her chest and radiating to her left arm. Her medical history was significant for hypertension and hyperlipidemia, managed with amlodipine and atorvastatin. She had no known history of coronary artery disease, heart failure, diabetes, or arrhythmias. She was a non-smoker, did not drink alcohol, and had an active lifestyle. Her family history was negative for cardiovascular diseases. Recently, she had been experiencing considerable emotional stress due to the sudden illness of a close family member, which she found particularly distressing.

On arrival, her vital signs showed a mildly elevated blood pressure of 150/85 mmHg, a heart rate of 92 beats per minute, respiratory rate of 20 breaths per minute, and oxygen saturation of 95% on room air. Physical examination revealed a mildly anxious but alert woman with no signs of respiratory distress, jugular venous distension, rales, or peripheral edema. A cardiovascular examination revealed a regular heart rate and rhythm without murmurs, rubs, or gallops. Lung sounds were clear, and the abdominal exam was unremarkable, with no tenderness or distention. Her neurological and musculoskeletal assessments were also normal.

Initial laboratory investigations included a complete blood count, comprehensive metabolic panel, cardiac biomarkers, and thyroid function tests. Results showed a white blood cell count of 10,500 cells/µL, likely due to stress, with normal renal and liver function. Her initial troponin I level was mildly elevated at 0.09 ng/mL (normal <0.04 ng/mL), consistent with myocardial injury. B-type natriuretic peptide (BNP) was elevated at 380 pg/mL, suggesting cardiac stress. A D-dimer level was within normal limits, reducing suspicion of pulmonary embolism.

**Figure 1:**
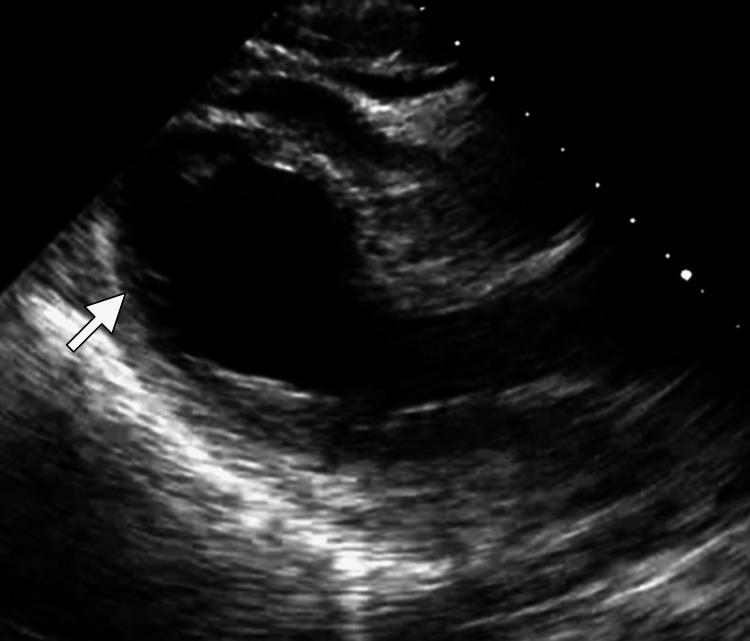
Three-chamber echocardiogram demonstrating hypokinesis of the mid to apical segments of the left ventricle (arrow) The apical and mid-ventricular regions showed reduced movement, characteristic of takotsubo cardiomyopathy, with preserved basal segment contractility. This echocardiographic pattern, often described as "apical ballooning," highlights the transient regional wall motion abnormalities associated with the condition.

The patient’s initial ECG revealed sinus tachycardia with ST-segment elevation in the anterior leads V2-V4 and subtle T-wave inversions in the lateral leads, raising concern for an acute anterior ST-elevation myocardial infarction (STEMI). To investigate further, a transthoracic echocardiogram was urgently performed, revealing apical ballooning and hypokinesis of the mid and apical segments of the left ventricle, with a reduced ejection fraction of 45% (Figure 1). These findings, combined with normal basal segment motion, suggested stress-induced cardiomyopathy, commonly referred to as TCM.

To confirm this suspicion and rule out obstructive coronary artery disease, the patient underwent coronary angiography. The angiogram demonstrated no evidence of significant coronary artery stenosis, which excluded an obstructive cause and reinforced the diagnosis of TCM. Differential diagnoses included ACS, given her chest pain, ECG changes, and troponin elevation; however, the absence of coronary artery disease on angiography and the echocardiographic pattern confirmed TCM as the most likely diagnosis.

Following the diagnosis, the patient was admitted to the cardiac care unit for close monitoring and management. She was started on a beta-blocker (metoprolol) and an ACE inhibitor to manage her blood pressure and support left ventricular function. Aspirin was initiated, though its benefit in TCM is uncertain, and diuretics were avoided as she had no signs of heart failure. During her stay, her ECG and troponin levels were monitored serially, showing no further elevation, and repeat echocardiography on day 4 demonstrated a slight improvement in left ventricular ejection fraction to 50%.

The patient’s hospital course was stable, without any episodes of arrhythmia or hemodynamic instability. She experienced progressive symptom relief and was discharged in stable condition with a referral for follow-up in the cardiology outpatient clinic. At her one-month follow-up visit, a repeat echocardiogram showed normalization of her left ventricular function with an ejection fraction of 60%, consistent with complete recovery from TCM. She was advised to continue her medications and seek regular cardiology evaluations, with a focus on stress management strategies to reduce the risk of recurrence.

## Discussion

This case of TCM highlights the unique clinical presentation, diagnostic challenges, and management considerations associated with this condition. Our patient, a 65-year-old woman with acute chest pain, ECG changes, and elevated cardiac biomarkers, initially presented in a manner suggestive of ACS. However, the absence of coronary artery obstruction on angiography, combined with the classic pattern of apical ballooning seen on echocardiography, confirmed the diagnosis of TCM [2-5]. This case underscores the importance of distinguishing TCM from ACS, particularly in postmenopausal women experiencing sudden emotional or physical stress. These factors, along with the transient nature of left ventricular dysfunction in TCM, are consistent with the current literature describing the typical presentation of this syndrome.

TCM predominantly affects postmenopausal women, likely due to hormonal and physiological changes that may influence stress response pathways and myocardial sensitivity to catecholamines. Studies indicate that up to 90% of TCM cases occur in women, with the majority in those aged 50 to 75. The relationship between stress and TCM, often termed "broken heart syndrome," is supported by our patient’s recent exposure to significant emotional distress [2,3]. Emotional triggers, such as grief or fear, and physical triggers, such as acute illness or surgery, are known precipitants of TCM, yet the exact mechanisms remain unclear. It is hypothesized that a surge in catecholamines during stressful events leads to a transient overload on the myocardium, resulting in the stunning of cardiac muscle fibers, particularly in the apical and mid-ventricular regions. This catecholamine hypothesis aligns with the pathophysiology often discussed in the literature, which emphasizes the central role of the autonomic nervous system and catecholamine toxicity in TCM development [2-4].

This case also raises awareness about the varied clinical presentations and ECG findings in TCM, which can often resemble those of an acute myocardial infarction (AMI). Our patient's initial ECG findings of ST-segment elevation in the anterior leads are common in TCM but are indistinguishable from STEMI. As such, coronary angiography remains critical in differentiating TCM from true myocardial infarction. In the literature, approximately 1-2% of patients presenting with suspected STEMI are ultimately diagnosed with TCM [1,5]. Unlike myocardial infarction, TCM usually presents with normal or near-normal coronary arteries, as was seen in this case. Echocardiography is also pivotal, as the classic pattern of apical and mid-ventricular ballooning, with hyperkinesis of the basal segments, provides strong evidence for TCM. Our case reflects these characteristic imaging findings and supports the echocardiogram as an essential tool in the diagnostic work-up.

In terms of management, the patient’s stable hospital course and subsequent recovery align with the generally favorable prognosis associated with TCM. Supportive therapy, including beta-blockers and ACE inhibitors, as prescribed in this case, are common approaches that aim to stabilize heart function and reduce myocardial oxygen demand. Although evidence on the long-term use of these medications in TCM is limited, studies suggest they may reduce the risk of recurrence and improve overall cardiac resilience [1-3]. Our patient’s recovery, with normalization of left ventricular function at follow-up, is typical, as most TCM patients regain normal cardiac function within a few weeks. However, serious complications, such as heart failure, arrhythmias, and even cardiogenic shock, have been documented in the literature and highlight the need for careful monitoring in the acute phase.

While TCM generally has a favorable prognosis, recurrence rates range from 2% to 5% and remain a concern for patients. Strategies to reduce recurrence, including lifestyle modifications and stress management, were emphasized in this case, aligning with recent literature advocating a holistic approach to patient care. Although TCM is reversible, its impact on quality of life, mental health, and future cardiovascular risk warrants comprehensive follow-up and patient education [2-4]. Our case reinforces the need for increased awareness and understanding of TCM, especially given its potential to mimic other cardiac emergencies. By adhering to the CARES guidelines, this case report contributes to the growing body of knowledge on TCM and supports ongoing research efforts to optimize diagnostic and therapeutic strategies for this unique and complex syndrome.

## Conclusions

In conclusion, this case of TCM underscores the importance of recognizing this syndrome as a distinct clinical entity that closely mimics ACS but differs significantly in its pathophysiology, management, and prognosis. This case highlights key elements of TCM, including its association with emotional stress in postmenopausal women, the characteristic pattern of transient apical ballooning, and the critical role of coronary angiography and echocardiography in diagnosis. While the prognosis of TCM is generally favorable, with most patients experiencing full recovery of cardiac function, early identification and differentiation from AMI are essential to avoid unnecessary interventions and optimize supportive management. Furthermore, our patient’s experience with TCM illustrates the need for ongoing follow-up and education on stress management, as recurrence remains a risk. This case contributes to the broader understanding of TCM, reinforcing the need for awareness in clinical practice and highlighting areas for future research into preventive strategies and long-term outcomes.
